# Dimethylsulfoniopropionate metabolism shapes microbial ecology and physiological adaptation during the austral winter in Southern Ocean sea ice and seawater

**DOI:** 10.1038/s41467-026-73596-x

**Published:** 2026-06-18

**Authors:** Z. Mayibongwe Buthelezi, Rian E. Pierneef, Oliver K. I. Bezuidt, M. Nello J. Gregori, Stephan Pesant, Daniele Iudicone, Libby Hanwell, Jonathan D. Todd, Thulani P. Makhalanyane

**Affiliations:** 1 Department of Biochemistry, Genetics and Microbiology, University of Pretoria, Pretoria, South Africa; 2 The School for Data Science and Computational Thinking, Stellenbosch University, Stellenbosch, 7600 South Africa; 3 DSTI/NRF SARChI in African Microbiome Innovation, Department of Microbiology, Faculty of Science, Stellenbosch University, Stellenbosch, 7600 South Africa; 4 European Bioinformatics Institute (EMBL-EBI), European Molecular Biology Laboratory, Wellcome Trust Genome Campus, Hinxton, Cambridge, CB10 1SD UK; 5 Stazione Zoologica Anton Dohrn, Naples, Italy; 6 School of Biological Sciences, University of East Anglia, Norwich, NR4 7TJ UK; 7 Quadram Institute, Rosalind Franklin Road, Norwich Research Park, Norwich, NR4 7UQ UK; 8 MOE Key Laboratory of Evolution and Marine Biodiversity, State Key Laboratory of Marine Food Processing and Safety Control, Frontiers Science Center for Deep Ocean Multispheres and Earth System & College of Marine Life Sciences, Ocean University of China, Qingdao, 266003 China; 9 Centre for Microbial Interactions, Norwich Research Park, Norwich, NR4 7TJ UK

**Keywords:** Environmental microbiology, Biogeochemistry

## Abstract

Dimethylsulfoniopropionate (DMSP) is a highly abundant marine organosulfur compound, with important roles in stress protection and climate-cooling gases production. Polar regions, particularly seawater and sea ice interfaces, are critical yet understudied DMSP cycling hotspots. Here, we reveal up to 38-fold higher DMSP concentrations in Southern Ocean sea ice versus seawaters, identifying sea ice as a concentrated reservoir of DMSP with implications for microbial stress tolerance and sulfur recycling. Eukaryotic algae harboring *DSYB* and *DSYE* genes were predicted to dominate DMSP production, but diverse and previously unidentified bacterial producers were also detected. This elevated abundance of algal biosynthetic genes likely underpins the higher DMSP concentrations in sea ice. Notably, DMSP catabolism, particularly the *dmdA* demethylase and *dddD* and *dddK* lyase genes, were more abundant than biosynthesis genes. Taken together, these findings reveal the widespread metabolism for DMSP cycling and underscore a dynamic reservoir and transformation hub influencing polar climate-cooling sulfur fluxes.

## Abstract (web summary)

Researchers reveal microbial metabolic pathways driving high levels of a climate-relevant sulfur compound in Antarctic sea ice and identify key microbes that may shape sulfur cycling and climate feedbacks in the Southern Ocean.

## Introduction

Understanding the ecology and physiology of microbial communities in extreme environments is critical for predicting ecosystem responses to global change^1,2^. In polar regions, such as the Southern Ocean (SO) marginal ice zone, seasonal freezing of seawater and thawing of sea ice imposes strong selective pressures due to rapid and substantial shifts in temperature, salinity and nutrient availability^3,4^. Previous studies have highlighted the key role of microbial communities in the biogeochemical cycling of carbon, nitrogen, and sulfur compounds in polar regions^5–8^. Consequently, these environments substantially contribute to atmospheric carbon dioxide uptake and the production and cycling of climate-active gases^9–11^. However, the mechanistic basis of microbial resilience and adaptation to poly-extreme environments, particularly within the SO marginal ice zone, remains poorly characterized.

Recent studies have explored microbial adaptation to sea ice conditions^12,13^, including the production of extracellular polymeric substances, the role of ice-binding proteins, and cold-adaptive gene clusters^14–17^. Nevertheless, critical knowledge gaps remain on the phylogenetic diversity and metabolic potential of microbial communities in the SO marginal ice zone, particularly during the austral winter season^12,18^. Notably, few studies have explored how SO microbial communities utilize labile compatible solutes, such as dimethylsulfoniopropionate (DMSP), to mitigate environmental stresses. Furthermore, there is also a lack of comprehensive molecular studies on microbial DMSP production and cycling in this region, despite DMSP’s proposed function in cryo- and osmo-protection^12,19,20^.

DMSP is one of Earth’s most abundant organosulfur compounds in marine ecosystems and is produced to millimolar intracellular concentrations in diverse organisms, including plants, algae, corals, and bacteria^21^. DMSP has important roles in chemotaxis, and protecting against salinity, cold, hydrostatic pressure, and oxidative stresses^22–26^. Once released into the environment, DMSP can be imported for its antistress properties or catabolised as a source of carbon, sulfur and/or energy^27,28^. Microbial DMSP catabolism can produce the volatile climate-cooling sulfur gases, dimethylsulfide (DMS) and methanethiol (MeSH) via DMSP cleavage or demethylation pathways, respectively, to impact global sulfur cycling and the climate^28–31^. Since DMSP synthesis and catabolism are often upregulated by environmental stress and/or DMSP availability, respectively^21,30^, sea ice formation in the SO marginal ice zones, likely influences microbial DMSP synthesis and cycling. Detailed studies combining DMSP measurement and molecular microbial ecology are urgently required to resolve the role/s of DMSP in environments transitioning from seawater to sea ice.

**Fig. 1:**
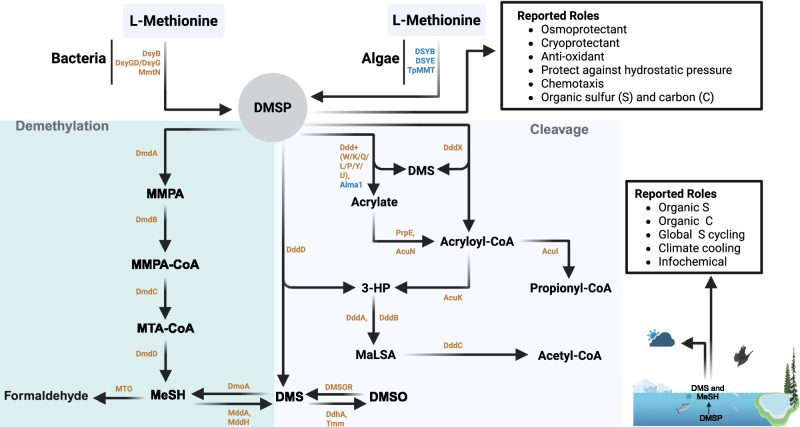
Pathways for bacterial and eukaryotic dimethylsulfoniopropionate (DMSP) biosynthesis and catabolism. DMSP biosynthesis begins with the metabolism of L-methionine. In algae, the key *S*-methyltransferase of the transamination pathway is encoded by *DSYB*, *DSYE,* and *TpMMT*. In bacteria, key *S*-methyltransferase enzymes in DMSP synthesis pathways are encoded by *dsyB*, *dsyG*, *dsyGD*, and *mmtN*. DMSP is degraded via two main pathways. In the demethylation pathway, *dmdA* demethylates DMSP to methylmercaptopropionate (MMPA), which is further processed by *dmdB*, *dmdC*, and *dmdD* to produce methanethiol (MeSH). In the cleavage pathway, diverse lyase enzymes, including bacterial Ddd and eukaryotic *Alma* family enzymes, cleave DMSP to generate dimethylsulfide (DMS) plus 3-hydroxypropionate (3-HP), acrylate, or acryloyl-CoA. 3-HP is subsequently transformed by *dddA*, *dddB*, and *dddC* to acetyl-CoA. Further transformations include the oxidation of DMS to dimethyl sulfoxide (DMSO) by DdhA and Tmm, the conversion of DMS to MeSH by DmoA, and the formation of DMS from H2S and MeSH, as well as H2S being a source of MeSH via MddA/MddH, and lastly the production of DMSO via DMSOR. The reported roles of DMSP and its gaseous catabolites, MeSH and DMS, are also described. Created in BioRender. Makhalanyane, T. (2026) https://BioRender.com/8d5lljt.

Identification of key DMSP synthesis and catabolic genes has enabled prediction of the organisms driving these processes in the environment via multi-omics analysis of community DNA, RNA and/or protein^21,32^ (Fig. 1). These genes include bacterial *mmtN* from the methylation pathway, and bacterial *dsyB* and *dsyG*/*dsyGD* or eukaryotic *DSYB*, *DSYE,* and *TpMMT* from the transamination pathway for DMSP synthesis^23,33–36^. DMSP can be demethylated by DmdA and the resulting methylmecaptopropionate (MMPA) product assimilated by ancillary Dmd enzymes for carbon, often yielding MeSH, which can be used as a sulfur source or further metabolized by MtoX to formaldehyde and assimilated^37^ (Fig. 1). Alternatively, DMSP can be cleaved by ten diverse DMSP lyase enzymes (Ddd or Alma family enzymes in bacteria/fungi or algae/corals) to yield DMS and a 3-carbon coproduct that is further processed by ancillary Ddd enzymes into central metabolism^28,29,38,39^. Moreover, DMSP lyases also cleave the DMSP-related metabolite dimethylsulfoxonium propionate (DMSOP)^40^, often abundant in marine environments, to produce dimethyl sulfoxide (DMSO) and 3-carbon coproducts^41^.

Here, we investigated the spatial diversity of DMSP, microbial communities, and their genetic capacity for DMSP synthesis and catabolism (Fig. 1) in SO seawater and sea ice during the austral winter of 2022. We hypothesized microbial communities in sea ice harbored enhanced DMSP synthesis and catabolic potential relative to those in seawater, as an adaptive strategy to cope with the environmental pressures in the SO marginal ice zone. This study provides important insights into the role of DMSP as a key microbial metabolite at the interface of ecology, stress physiology, biogeochemical cycling, and climate regulation in polar marine ecosystems.

## Results

### DMSP standing stock concentrations in seawater and sea ice core samples

**Fig. 2:**
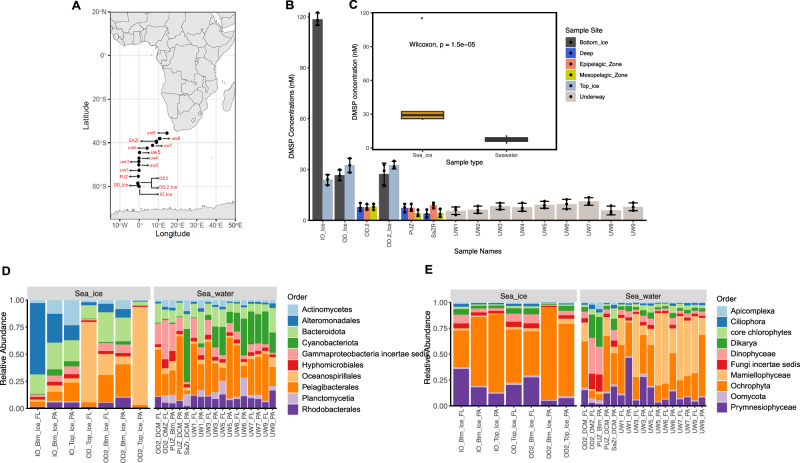
Presents sampling cites co-ordinates, DMSPt concentrations and relative abundance of microbial communities in SO sea ice and seawater samples. **A** Map of the SCALE cruise track and sampling stations where seawater and sea ice samples were collected to assess in situ DMSP concentrations and associated microbial communities. This figure was created in BioRender. Makhalanyane, T. (2026) https://BioRender.com/hthyqt1. **B** DMSP standing stock concentrations derived from the average of three biological data points and error bars show standard deviation between data points (*n* = 3). The sample site information is indicated by a color and described in a key. **C** Box plots represent the distribution of the DMSP concentrations with Wilcoxon test showing significant differences between sea ice and seawater samples *p-value* = 1.5e-05. The central line indicates the median, the box bounds represent the 25th and 75th percentiles (interquartile range, IQR), and the whiskers extend to the minimum and maximum values. **D** Relative abundance of dominant bacterial taxa, and **E** dominant eukaryotic taxa identified in seawater and sea ice samples based on metagenomic analysis. For these figures source data are provided as a Source Data file.

The particulate plus dissolved DMSP concentrations (DMSPt) were measured in seawater and sea ice samples collected during the Southern oCean seAsonaL Experiment (SCALE) austral winter expedition (11^th^ of July to 31^st^ of July 2022) aboard the RV *SA Agulhas II* (Fig. 2A, Supplementary Fig. S1). The station labels SaZr and PUZ represent the sub-Antarctic zone and polar frontal zone, respectively. Moreover, the OD and IO sea ice stations correspond to open drift pancake and consolidated young sea ice, respectively (Fig. 2A). DMSPt concentrations in seawater ranged between 3 nM and 11 nM (*n* = 18 samples). Samples from the ship’s underway (UW) system and those collected at 50 m and within the epipelagic zone (EPZ) exhibited higher DMSPt levels compared to samples from deeper layers, particularly those from the 1000 m mesopelagic zone (MSP) and 2000 m deep layer (Fig. 2B). DMSPt levels were consistent with previous studies of sea water samples^35,42,43^. Notably, sea ice DMSPt concentrations were always more variable (24 nM to 115 nM, *n* = 6 samples), but 2 to 38 times higher than those in seawater samples (Fig. 2C), which was consistent with previous reports^12,20,44^. As a compatible solute^25^, the markedly higher DMSPt levels observed in sea ice suggest an increased cellular quota for DMSP, consistent with its crucial role in microbial survival under cold and hypersaline conditions^45–48^.

The mechanisms underpinning DMSP’s function as both an osmoprotectant and cryoprotectant are essentially the same: enhanced synthesis and/or import (particularly in non-DMSP producers) and accumulation within cells. This allows DMSP to act as an osmolyte, preventing the efflux of water molecules into the highly saline extracellular environment, such as in brine channels^25^. As with other osmolytes ^25,48^, DMSP likely also modulates intracellular and sometimes extracellular environments to reduce intracellular ice formation and prevent cellular damage. The high microbial demand for DMSP to survive sea ice conditions likely reduces its catabolism for sulfur and carbon, thereby contributing to its elevated concentrations in sea ice. This is consistent with the net higher DMSPt levels in sea ice compared to seawater, where salinity conditions are less extreme and there is reduced pressure on microbes to produce and retain high levels of DMSP. Note, it is also possible that the raised DMSPt concentrations in sea ice were due to higher cell abundance and/or decreased DMSP degradation rates (not measured here), but the latter seems unlikely considering the metagenomic analysis below.

The top section of all sampled ice cores showed similar DMSPt concentrations (Fig. 2B). However, there were ~ 4-times higher DMSPt levels in the bottom ice cores retrieved from IO compared to those from OD and OD.2 samples. This higher DMSPt concentration in IO, a young sea ice sample, aligns with findings of Trevena and Jones^44^, which showed that the newly consolidated sea ice microbes, among other factors, are exposed to higher light levels that enhances productivity and promotes DMSP biosynthesis. Note, only austral winter samples were analysed and DMSP flux is strongly influenced by space and time^49^, thus, these phenomena may not be representational of the other SO regions and seasons. Nevertheless, we hypothesize that the varying DMSPt levels, observed between different austral winter sea ice samples and compared to seawaters, were likely due to differences in microbial communities, their ability and requirement to produce, store and/or cycle DMSP, and/or sample biomass. Unfortunately, no measurement of sample biomass nor of DMSP production and catabolic rates was conducted, which could have informed on the observed differences in DMSPt levels. However, comprehensive metagenomic analyses supported changes in microbial communities and their metabolic profiles as plausible explanations for the observed differences in DMSP levels.

### Composition of the most abundant microbial communities in the samples

Metagenomic analyses of the austral winter sea ice and seawater samples was conducted to gain insights on microbial DMSP production and cycling, the variability in DMSPt standing stock concentrations and to address a key knowledge deficit on microbial communities and their distribution in the SO during this time of the year. Initially, the composition and abundance of microbial communities were analysed from metagenomic sequence data, retrieved from > 3.0 µm and 0.2 – 3.0 µm size fractions. These size fractions are generally apportioned to particle-attached bacteria/large phytoplankton and free-living picophytoplankton/bacterioplankton, respectively^50^. Analyses of the Bray Curtis dissimilarity matrix revealed a clear divergence in microbial community composition between taxa inhabiting seawater and sea ice ecosystems (Supplementary Fig. S2). Here, we report on the top ten most abundant taxa across the samples.

There was considerable variability in bacterial communities within the seawater samples from different depths (Fig. 2D). Cyanobacteriota were generally predominant in underway surface samples compared to EPZ samples (except for the SaZr). These taxa were scarce in samples from the MSP, deep-water layers, sea ice, and the EPZ of colder waters (PUZ and OD2), revealing a clear ecological preference for photic, relatively warmer SO waters by Cyanobacteriota. Inversely, the relative abundances of Gammaproteobacteria were 1.82-fold greater in MSP and deep samples combined compared to EPZ samples, consistent with previous global ocean surveys^18^. More substantial differences were observed between seawater and sea ice microbial communities. Indeed, Cyanobacteriota and Pelagibacterales (SAR11 clade) were prominent in the seawater bacterial community (16.0% and 33.8%) compared to sea ice samples (1.88% and 13.2%).

In sea ice, Alteromonadales (particularly in IO samples) and Oceanospirillales (particularly in the top section of OD and OD2 samples) were generally far more abundant, comprising 8.21% and 41.9% of the bacterial community compared to 1.92% and 3.42% in seawater, respectively. It was interesting that Oceanospirillales, known as prominent DMSP consumers^6^, were distinguishably more abundant within the top ice section of OD/OD2 samples, where DMSPt concentrations were considerably lower than in IO bottom ice samples. Conversely, the significantly higher abundance of Alteromonadales in the bottom half of IO ice samples was consistent with the far higher DMSPt concentration in these compared to other sea ice samples, since these bacteria were previously reported to play a crucial role in DMSP production in marine sediments^35^. However, higher DMSP concentrations are primarily attributed to algae, especially those categorized as high accumulators^34^, which were also enriched in the IO bottom ice samples (see below) and were likely the major DMSP producers in this sample.

There were many other potential DMSP-producing bacterial taxa within the seawater and sea ice samples. These taxa include Rhodobacterales (9.10% and 4.20%), Bacteroidota (12.8% and 16.9%), Actinomycetes (3.56% and 6.49 %), and Gammaproteobacteria (10.8% and 4.40%), respectively, consistent with previous findings^21,33,51^. Moreover, some Cyanobacteria (particularly, *Oscillatoria* spp.) also produce DMSP through the *dsyG/dsyGD* gene products^34^. However, there was no obvious correlation between the abundance of these taxa and observed DMSPt concentrations, and DMSP production is far from being universal in these taxa. Note, many of the most abundant bacterial taxa in the samples, e.g., Pelagibacterales, Rhodobacterales, and other detected proteobacteria, are known for their ability to import and catabolize DMSP via DMSP cleavage and/or demethylation, which impacts DMSPt levels^41^.

Moreover, the relative abundance of key eukaryotic taxa also varied within the seawater and sea ice, with respective relative abundances of 30.4% and 1.99% for Mamiellophyceae, 30% and 66.2% for Ochrophyta, 12.4% and 15.0% for Prymnesiophyceae, 6.47% and 3.73% for Dikarya, and 7.69% and 4.67% for Dinophyceae (Fig. 2E). Within seawater samples, the prominent Ochrophyta, Mamiellophyceae and Prymnesiophyceae taxa were far more abundant in surface water samples, compared to fungi, Dinophyceae and Dikarya that were more abundant in MSP and deep water samples. The elevated DMSPt concentrations observed in sea ice versus seawater were likely attributable to dominance in the former of Ochrophyta and Prymnesiophyceae taxa previously categorized as high DMSP accumulators^34^. Furthermore, the higher relative abundance of these Prymnesiophyceae (with much larger cell volumes and DMSP concentrations than DMSP-producing bacteria^21,34^) in bottom IO sea ice (both FL and PA fractions) versus the top samples, implicated these algae as the major source of higher DMSPt levels in the bottom layer, with DMSP-producing bacteria, like Alteromonadales, having a less significant role. However, as previously noted, it is difficult to accurately predict the organisms that are producing and cycling DMSP from taxonomy data alone^21^. Assays of DMSP synthesis and catabolic gene abundance and/or expression via multi-omics would allow for more robust prediction of the key microbes driving SO DMSP production and cycling.

### The abundance of genes related to DMSP production and cycling

**Fig. 3:**
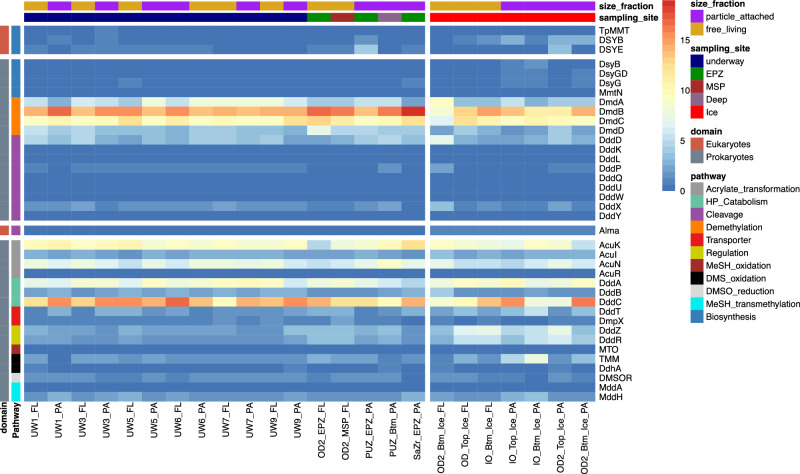
Normalized relative abundance of genes involved in bacterial and eukaryotic DMSP production and cycling pathways. The relative abundance of these genes were normalized to *recA* (bacteria and 0.2 – 3 μm algae) and *actin* (>3 μm algae). The Domains of life and metabolic pathways individual genes belong to are indicated by the colors at the left of the data plots and are detailed in the keys. Annotations above the data plots indicate the sampling site and size fraction by different colors which are detailed in the keys. For this figure source data are provided as a Source Data file.

In silico analysis of metagenomic sequence data was conducted to study the relationship between the genetic potential to synthesize and catabolise DMSP, microbial taxonomy and DMSPt concentrations. A hidden Markov model (HMM) search, similar to Teng, et al.^52^, was used to identify DMSP synthesis and catabolic gene abundance and diversity within the sea ice and seawater samples. Subsequently, the relative abundance of genes for DMSP production and degradation in bacteria and 0.2–3 μm algae were normalized to *recA* and >3.0 μm algal genes to *Actin*, to estimate the relative abundance of a DMSP gene within the community, similar to^34,52^ (Fig. 3). As indicated in the Fig. 3 legend, the relative abundance of these genes ranged from 0 to ~17% of the total microbial community.

### Algal DMSP production

**Fig. 4:**
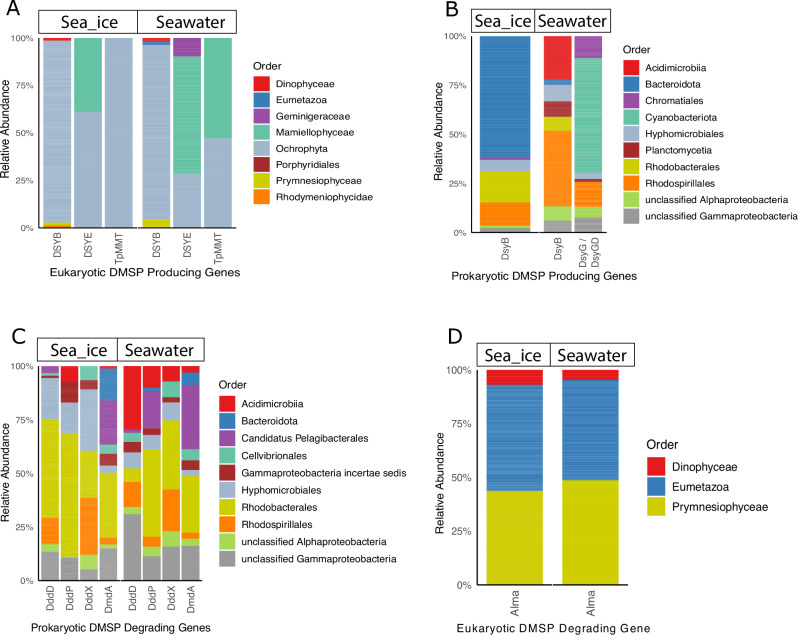
Taxonomic distribution of DMSP-related genes/enzymes in Southern Ocean sea ice and seawater. **A** Dominant eukaryotic taxa (order level) encoding DMSP biosynthesis genes. **B** Dominant prokaryotic taxa (order level) encoding DMSP biosynthesis genes. **C** Dominant prokaryotic taxa (order level) encoding DMSP degradation genes.** D** Dominant algal taxa (order level) encoding DMSP degradation genes. For these figures source data are provided as a Source Data file.

Current literature considers marine algae as the major global DMSP producers in Earth’s photic oceans. Most known DMSP-producing algae contain *DSYB*, *DSYE*, and/or *TpMMT* genes and their presence in metagenomic data can be used to infer key environmental DMSP producers^21,23,34^. All SO sea ice and seawater samples contained these algal DMSP synthesis genes, which were predicted in 1.76% eukaryotic taxa across the samples, comprising *DSYE* (0.83%), *DSYB* (0.75%), and *TpMMT* (0.18%) (Fig. 3). *DSYE*, the most abundant eukaryotic DMSP synthesis gene, exhibited far higher relative abundance in seawater compared to sea ice samples, where it comprised 33.03% and 19.81% of the detected DMSP synthesis genes, respectively (Supplementary Fig. S3). Sea ice and seawater *DSYE* genes were predicted to be from Ochrophyta (Bacillariophyceae and Pelagophyceae lineage) and Mamiellophyceae (class Chlorophyta), consistent with the findings by Wang et al.^34^. Interestingly, *DSYE* was also predicted within the Geminigeraceae family in seawater samples (Fig. 4A), further expanding the known diversity of microbial groups with potential for DMSP biosynthesis.

*DSYB* was the second most abundant eukaryotic DMSP synthesis gene across all the samples (Fig. 3) but was the most abundant in sea ice samples. In sea ice samples, *DSYB* comprised 36.17% of detected DMSP synthesis genes versus 13.63% in seawater samples (Supplementary Fig. S3). In both sea ice and seawater, *DSYB* was predominantly predicted in Ochlorophyta (Bacillariophyceae lineage), and to a lesser extent, some Dinophyceae and Prymnesiophyceae (Fig. 4A). This is consistent with previous studies in the marine environment where these taxa were identified as important DMSP producers^21,23^. Eukaryotic *DSYB* was also identified, at much lower levels, in some seawater Eumetazoa and sea ice Rhodymeniophycidae.

*TpMMT* was the least abundant eukaryotic DMSP synthesis gene, but was on average slightly more abundant in seawater (6.62%) versus sea ice (3.98%) samples (Fig. 3; Supplementary Fig. S3). Sea ice *TpMMT* was exclusively from Ochrophyta (Bacillariophyceae lineage), but in seawater this gene was predicted in Mamiellophyceae as well as Ochrophyta. The microbial taxa harboring *TpMMT* in these environments are consistent with previous findings^21,34^.

Together, these data further supports that algae with *DSYE* and *DSYB* were major drivers of the observed DMSPt levels in SO seawater and sea ice, and that algae are the major DMSP producers in marine photic waters and sea ice^44,53^. Unsurprisingly, algal *DSYB*, *DSYE,* and *TpMMT* genes on average were more abundant in photic than aphotic (MSP and deep) samples, probably owing to the phototrophic lifestyle of their host. However, detection of *DSYB*, *DSYE,* and *TpMMT* sequences in the MSP and deep samples highlights their ubiquity in the SO and unexpected potential importance in aphotic systems. Detection of *DSYB*, *DSYE,* and *TpMMT* in these unexpected water layers may have resulted from the deeper SO austral winter mixing^54^, isolation of sinking environmental DNA from dead or inactive cells, or highlight the functional importance of these enzymes across different depths of the water columns. Further work involving metatranscriptomics or metaproteomics may have given a better indication on the active organisms responsible for DMSP production in the photic and aphotic systems.

### Bacterial DMSP Production

A diverse group of bacterial taxa in SO seawater and sea ice possessed the key marker *dsyB* and *dsyG/dsyGD* genes for bacterial DMSP biosynthesis, hinting that prokaryotes contributed to the observed DMSPt concentrations (Fig. 3). No *mmtN* genes were detected, consistent with previous studies showing that this gene is largely niche specific and restricted in global distribution^35^. These DMSP synthesis genes were predicted in 1.36% of the bacterial community, with *dsyG, dsyGD,* and *dsyB* comprising 0.53%, 0.49%, and 0.33%, respectively (Fig. 3). In SO sea ice, *dsyB* was more abundant compared to seawater, comprising 13.48% and 9.66% of the detected DMSP synthesis genes in the respective samples (Supplementary Fig. S3). Unfortunately, we still know very little about the environmental drivers of *dsyB* gene abundance in diverse environments, yet Curson et al.^55^ showed *dsyB* transcription could be upregulated by low nitrogen and raised salinity levels, the latter of which may be relevant in hypersaline environments like sea ice. Consistent with previous findings^33,56^, bacteria with *dsyB* in both SO seawater and sea ice were predicted to be Bacteroidota, Hyphomicrobiales, Rhodobacterales, Rhodospirillales, and unclassified Alpha- and Gamma- proteobacteria (Fig. 4B). Additionally, *dsyB-*containing Acidimicrobiia were detected exclusively in seawater samples, further highlighting the spatial variability in the distribution of taxa carrying *dsyB* across the SO.

Surprisingly, *dsyG* and *dsyGD* comprised 19.66% and 13.22% or 17.4% and 13.22% of DMSP synthesis genes in seawater or sea ice samples, respectively (Fig. 3, Supplementary Fig. S3). This study marks an important report of *dsyG* or *dsyGD* in a marine environmental sample, with these genes being largely absent in the *Tara* Oceans dataset^34^. Although, the taxonomy of *dsyG/dsyGD* in sea ice samples could not be assigned to any known bacteria (due to *E-value* cutoff), in seawater, these genes were predominantly predicted in Cyanobacteria consistent with previous finding by Wang et al.^34^ (Fig. 4B). Moreover, *dsyGD* was also identified at lower abundance in Chromatiales, Hyphomicrobiales, Planctomycetia, Rhodospirillales and unclassified Alpha- and Gamma- proteobacteria. These findings expand current understanding of *dsyGD* in these taxa and provides insights on the distribution of this gene across phylogenetically diverse microbial lineages in the Southern Ocean.

Bacterial DMSP synthesis genes were only more abundant than *TpMMT* when compared to their algal counterparts (Fig. 3, Supplementary Fig. S3). The abundance of taxa containing DMSP synthesis genes in this study were far higher than previously reported, e.g., 0.141% for *dsyB* in non-polar samples^56^. These data, with the inferred relatively high abundance of DMSP producers in SO samples from taxonomy, highlights a probable strong requirement for DMSP in microorganisms that thrive in the SO marginal ice zone. Notably, microorganisms (both bacteria and algae) that were predicted to produce DMSP were also amongst the most abundant taxa in both seawater and sea ice core samples (Fig. 2D–E), implying the crucial role of DMSP in shaping the ecology and physiological traits of key microbial taxa in the SO. However, the prominence of these taxa is not necessary a reflection of their contributions to the observed absolute concentrations of DMSPt. These groups include both previously identified, well characterized DMSP producers as well as those not previously reported to produce it. As previously demonstrated^21^, DMSP produced via these gene products likely played a significant physiological role in enabling microbial adaptation to the fluctuating and stressful conditions of the SO marginal ice zone.

### DMSP catabolism

For organisms that cannot produce DMSP, this compound must first be imported from dissolved DMSP present in the water column or sea ice e.g., via *dddT*^57^ and *dmpX*^58^ in bacteria. These DMSP transporter genes exhibited varied relative abundances, *dddT* and *dmpX* were predicted in 2.28% and 0.98% of taxa across all samples (Fig. 3). DMSP catabolic genes and those encoding transcriptional regulators of DMSP catabolism *dddR* and *dddZ*^59,60^ were represented in 2.80% and 3.34% taxa across the SO samples, respectively.

Microbial DMSP catabolic genes of the cleavage and demethylation pathways^29^ were identified in all samples. The bacterial DMSP demethylase gene *dmdA* was the single most abundant primary DMSP catabolic gene across the samples, which was present in 5.35% taxa (Fig. 3). Moreover, this gene comprised 46.15% and 39.08% of the detected DMSP catabolic genes in seawater and sea ice, respectively (Supplementary Fig. S4). The SO seawater and sea ice *dmdA* genes were from Acidimicrobiia, Candidatus Pelagibacterales, Rhodobacterales, Cellvibrionales, Bacteroidota and unclassified alpha- and gamma- proteobacteria (Fig. 4C) consistent with previous reports^29,38,61^. Pelagibacterales, which were abundant in the SO samples have been shown to utilize DMSP demethylation as a primary means of acquiring sulfur and as a carbon source for their growth and metabolism^62–64^. These data imply that bacterial DMSP demethylation is the major DMSP catabolic pathway in SO seawater and sea ice, which is likely important for microbial assimilation of sulfur and carbon from DMSP^29,65^.

Although the DMSP lyase genes were each singularly less abundant than *dmdA*, collectively the *dddD/K/L/P/Q/U/W/X/Y* and *Alma* genes were more abundant in both seawater and sea ice samples, being predicted in 6.22% and 0.41% of bacteria and algae, respectively (Fig. 3). Moreover, *dddD*, *dddX,* and *dddP* were the most abundant DMSP lyase genes, predicted in 3.50%, 1.51% and 0.76% of bacterial taxa, respectively, with *dddY* being the least abundant in 0.002% taxa (Fig. 3), consistent with previous reports^52,65^. Note, *dddD*, *dddX* and *dddP* accounted for similar 29.12%, 12.43% and 6.57% of the detected DMSP catabolic genes in seawater and 30.77%, 13.97% and 6.13% in sea ice, respectively (Supplementary Fig. S4). These genes were largely associated with Candidatus Pelagibacterales, Rhodobacterales, Rhodospirillales, Hyphomicrobiales, Acidimicrobiia, unclassified Alpha- and Gamma- proteobacteria (Fig. 4C). However, there was some niche specialization, with Acidimicrobiia *dddD* and *dddX*, and Candidatus Pelagibacterales and Rhodospirillales *dddP* being exclusive to seawater samples. Unlike, the *dsyB* containing Acidimicrobiia which was detected exclusively in seawater (Fig. 4B), these taxa containing *dmdA* and *dddP* genes were present in both seawater and sea ice samples (Fig. 4C). These results imply that sea ice Acidimicrobiia primarily rely on importing and utilizing DMSP as a source of sulfur and/or carbon for assimilation and likely utilize DMSP-independent mechanisms to cope with sea ice restricting conditions.

The algal Alma family DMSP lyases^66^ were also identified in sea ice and seawater samples, comprising 7.49% and 1.55%% of the detected DMSP degrading genes, respectively (Fig. 3). Their protein products were predicted in Dinophyceae, Eumetazoa and Prymnesiophyceae (Fig. 4D) which has been highlighted previously^67^. However, the mechanism of *Alma* genes in Eumetazoa is not well known, and further investigation is required to understand how this group utilizes DMSP in the SO seawater and sea ice ecosystems. Algal DMSP cleavage is proposed to serve as an important antioxidation, predator deterrent and/or carbon assimilation mechanism^22,26,28,68^. Note, since the Ddd and Alma enzymes can cleave DMSP as well as DMSOP (not measured here), it is possible these stressful SO environments, like marine sediments^41^, are also hotspots for production and/or catabolism of both compounds. However, there are currently very few in situ measurements of DMSOP, which are required to better understand the global importance of this recently identified sulfur compound^40,41^. This study takes important steps to uncover the distribution and significance of microbial DMSP cleavage (bacterial and algal) and demethylation (bacterial) in SO seawater and sea ice environments.

### Downstream catabolism of DMSP catabolites

After primary DMSP cleavage or demethylation, the resultant catabolites including methylmercaptopropionate (MMPA) and MeSH for demethylation and DMS, acrylate, 3-HP and acryloyl-CoA for cleavage, can be further catabolised (Fig. 1). The genes for this secondary catabolism were highly abundant in sea ice and seawater (Fig. 3), including *acuI* predicted in 2.48%, and *dddA, dddB* and *dddC* in 7.98, 2.23 and 13.04% of bacteria, respectively. These genes allow for the transformation of the DMSP cleavage catabolites acrylate (*acuI)* and 3-HP (*dddABC*) into central metabolism for assimilation^69^. Similarly, *dmdB* and *dmdC* genes, which encode for the degradation of the DMSP demethylation catabolite MMPA^29^, were also highly abundant in both sea ice and seawater samples, being predicted in 14.32 and 10.16% of bacterial taxa, respectively. The abundance of these ancillary *dmd* genes is likely due to their broader functionality which allows them to facilitate various reactions outside DMSP metabolism^70^. Note, the DMSP demethylation pathway gene *dmdD* (detected in 3.73% of bacterial taxa), whose product facilitates carbon assimilation from MMPA and MeSH liberation, showed a relatively lower abundance compared to *dmdB* and *dmdC* (Fig. 3).

The DMSO reductase (DMSOR) genes^71^ and *mddA/H*^72,73^, whose enzyme products yield DMS from DMSO and hydrogen sulfide/MeSH, respectively, were also abundant in these samples. These genes were present in 1.23 % (DMSOR) and 0.07/1.95 % (*mddA/mddH)* of bacterial taxa, but were less abundant than the DMSP lyase genes. Unsurprisingly, *mddA* was far less abundant than *mddH*, consistent with this gene being more associated with terrestrial environments^73^. *MddH* was notably more abundant in the SO marginal ice zone, perhaps contributing to enhanced production and emission of DMS which are characteristic of polar regions^74,75^.

Additionally, DMS oxidation potential was identified in all samples, primarily via *tmm* (trimethylamine monooxygenase) and *ddhA* (DMS dehydrogenase) whose products yield DMSO^76,77^. However, while these data are robust, they only demonstrate the potential of SO microbes to facilitate DMSP and related cycling, and further evidence of gene expression or translation profiles are necessary, together with detailed process measurements to support hypotheses.

### High quality MAGs containing genes linked to DMSP production and degradation

**Fig. 5:**
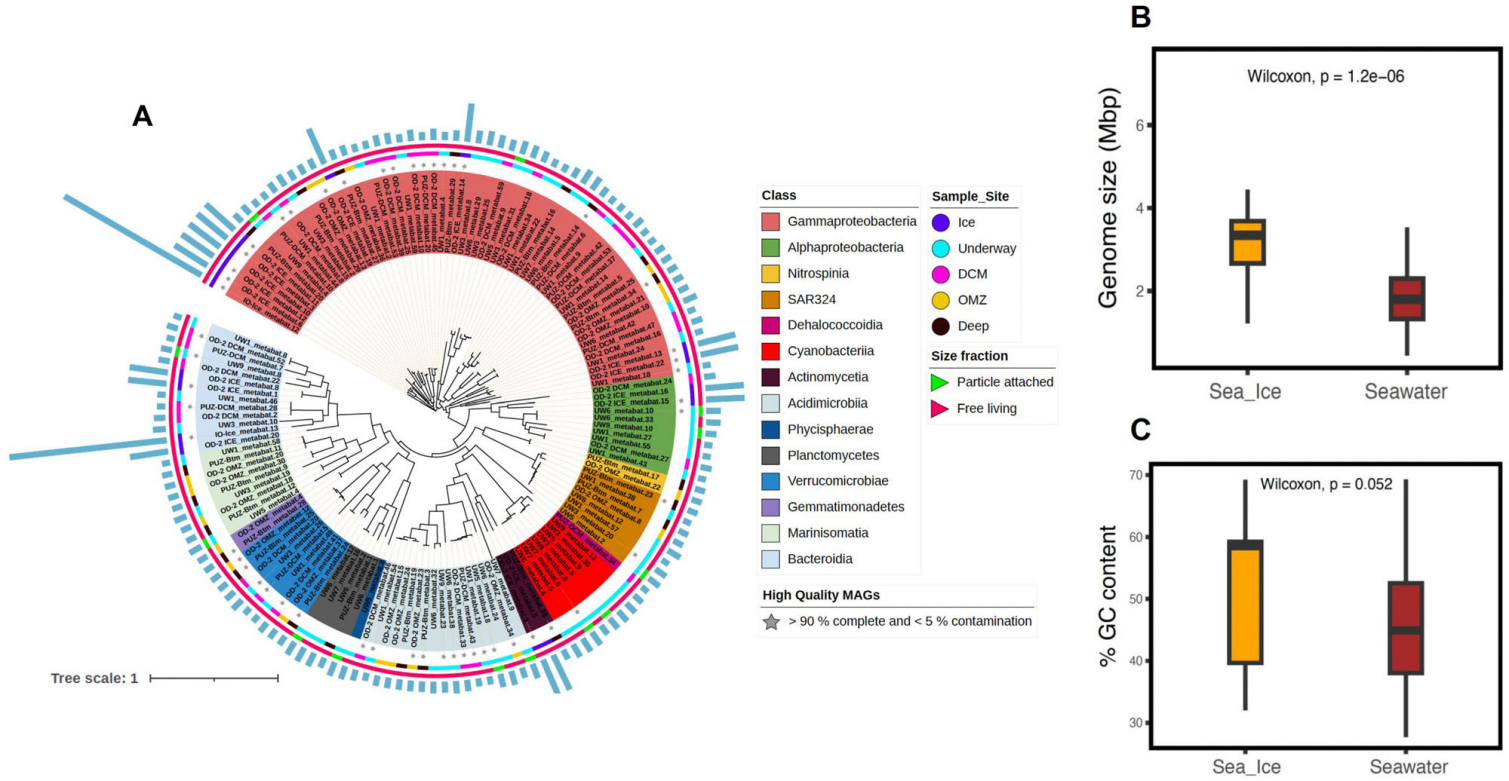
Phylogenetic diversity of the reconstructed genomes, and their genome sizes and GC content differences between sea ice and seawater samples. **A** Bacterial metagenome-assembled genomes (MAGs) at the class level. Asterisks denote high-quality MAGs. Annotations show the sample origin and size fraction; accompanying bar plots indicate DMSPt concentrations in corresponding samples. Box plots is the comparison of genome size distributions **B** and GC content **C** between MAGs derived from seawater and between sea ice samples, where the central line indicates the median, the box bounds represent the 25th and 75th percentiles (interquartile range, IQR), and the whiskers extend to the minimum and maximum values. The Wilcoxon test was used for statistical analyses for both genome size and GC content in (**B**) and (**C**) was derived from 148 MAGs, respectively. For these figures source data are provided as a Source Data file.

**Fig. 6:**
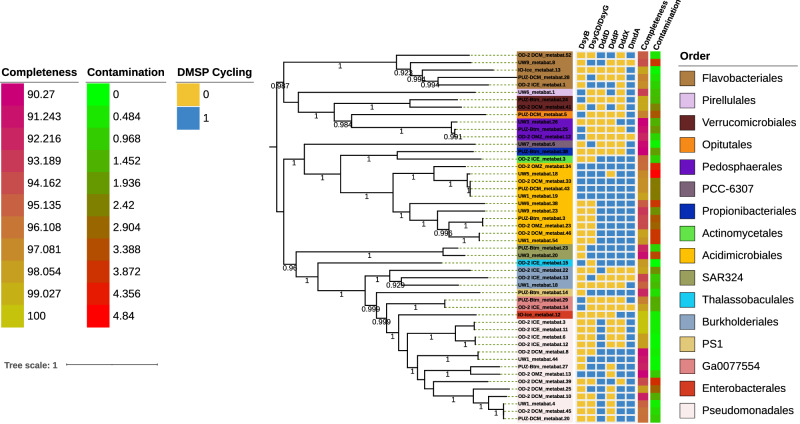
Maximum likelihood phylogenetic tree of 50 bacterial high quality MAGs at the order level. The heatmap indicates the presence or absence of putative DMSP biosynthesis and degradation genes. Tree topology was supported by 1000 bootstrap replicates; only bootstrap values between 90% and 100% are shown. Outer color gradient bars represent genome quality based on completeness and contamination metrics. For these figures source data are provided as a Source Data file.

Metagenomic data was utilized to reconstruct 148 medium (98) to high (50) quality metagenomic assembled genomes (MAGs), providing insights into the diversity of bacterial genomes associated with these SO samples (Fig. 5A). Subsequently, high quality bacterial MAGs were selected and analysed for the presence of genes linked to DMSP production and catabolism (Fig. 6). In these bacterial genomes, *dsyB* and *dsyG/dsyGD* for DMSP production were mostly in Pseudomonadales, Acidimicrobiales and Flavobacteriales taxa. Interestingly, *dsyG/dsyGD* and *dsyB* were frequently both co-encoded within the same Acidimicrobiales genomes, suggesting a potentially important role for DMSP biosynthesis in these taxa. Furthermore, 90 % of the assembled genomes also harbored *dmdA* and 68, 54, and 44 % contained *dddD*, *dddX,* and *dddP* genes, respectively (Fig. 6). These genes were observed to be orthologous and shared across genomes that spanned diverse taxonomic groups which hinted that these taxa may have acquired DMSP cycling genes from the environment. A phylogenetic reconstruction using DddP proteins acquired from 38 genomes indicated that these were likely acquired through horizontal gene transfer (HGT) events (Supplementary Fig. S5). This implies that HGT promotes the dissemination of key metabolic genes to allow microbes access to nutrient sources that would increase their ability to compete within the environment^78^. In fact, most genomes with DMSP synthesis genes also contained catabolic potential (84.62%), demonstrating the importance of being able to respond to environmental changes and utilize DMSP for its antistress properties and/or as a sulfur and/or carbon source, a phenomenon that occurs in many bacteria^55^. Some genomes within the Acidimicrobiia, Thalassobaculales and Pseudomonadales groups contained marker genes for both DMSP demethylation and cleavage pathways. However, it was not possible to predict the partitioning through DMSP demethylation and cleavage pathways from this study, which was likely under coordinated kinetic regulation based on the specific microbial requirement for DMSP^38,79^. These findings further reveal the uniqueness and phylogenetic diversity of bacterial community capable of DMSP production and degradation in the SO.

Microbial genome size and guanine and cytosine (GC) content. The 148 SO MAGs were analysed for their genome size (Fig. 5B) and GC content (Fig. 5C), between seawater and sea ice core samples. Genome size and GC content are important parameters that reflect microbial metabolic complexity and ecological signatures^80,81^. The sea ice microbial communities possessed relatively higher average GC content (mean average ∼59%) and significantly larger genome size (mean average ∼3.7 Mbp) values compared to those in seawater with ∼45% GC content and ∼1.9 Mbp genome size. These findings are consistent with previous findings by Ngugi et al.^82^ showing that polar microbes have high GC content and larger genome size which is essential for thriving in these extreme and complex environments. Additionally, these results highlight the possible selective pressures that are shaping microbial adaptation and evolution, driven by metabolic complexities within the distinct environments of sea ice and seawater.

## Discussion

This study measured DMSPt standing stock concentrations, the relative abundance of genes involved in DMSP production and catabolism, and further analysed the microbes likely driving these pathways in the SO seawater and sea ice. DMSPt concentrations in sea ice samples were 2–38 times higher than those in seawater. Given its role as a compatible solute^26,48^, it is reasonable to infer that microbes in sea ice may have an increased demand for DMSP, likely for cryoprotection and osmoprotection. Moreover, the observed variations in DMSPt concentrations were often supported by shifts in the relative abundance of microbial communities, as well as marker genes encoding for DMSP production and degradation between the seawater and sea ice samples. Consistent with findings of Teng et al.^52^, our data implied eukaryotic algae as the major DMSP producers in SO sea ice and sea water, with the combined abundance of *DSYB*, *DSYE,* and *TpMMT* genes comprising ~56.62% of detected DMSP synthesis genes. Although, the relative abundance of heterotrophic bacteria with *dsyB* and *dsyG/dsyGD* was lower (1.36%), they likely had an important contribution. Diverse algae with *DSYB* were associated with the higher DMSPt levels in sea ice, but those with *DSYE* were clearly dominant in seawater. Interestingly, the combined eukaryotic and prokaryotic DMSP synthesis gene abundance was still far less than that for DMSP degradation, particularly *dmdA* and *dddD*. This implied a significant portion of DMSP may serve as an important microbial sulfur and/or carbon source in these environments.

The taxonomic classification of DMSP synthesis and catabolic genes revealed associations with a wide range of microbial groups, including both the most and least abundant taxa along the transect. This highlighted the broad distribution of these genes across the SO taxa and provided insight on the functional importance of DMSP for diverse microbial communities in both seawater and sea ice. Among the lower-abundant taxa, this study provides evidence of the genetic capacity for DMSP production in genomes from the order Acidimicrobiales. This suggests a broader and previously unrecognized diversity of microbes involved in SO DMSP production and cycling. This study further advanced our understanding and revealed important insights in the SO ocean microbes, aligning with previous findings showing that approximately 80% of microbial metabolic potential in this region remains undescribed Sunagawa et al.^18^.

Altogether, this study provides compelling evidence that the Southern Ocean marginal ice zone is a critical hotspot for global sulfur cycling and climate regulation through DMSP production and its cycling potentially releasing both DMS and MeSH. While the absence of physical parameter data, process rate measurements, and expression profiles of key DMSP synthesis and catabolic genes/proteins limits the extent of our conclusions, our findings establish a strong foundation for future integrative studies. Addressing these gaps will be essential to fully resolve the role of the Southern Ocean in regulating biogeochemical sulfur fluxes and their influence on the global climate system.

## Methods

### Water and ice core sampling

Seawater and ice core samples were collected during the Southern oCean seAsonaL Experiment (SCALE) austral winter cruise (11^th^ of July to 31^st^ of July 2022) aboard the RV *SA Agulhas II*. Surface water samples were collected using the underway system at nine stations, and water column samples were collected at three stations using a CTD rosette equipped with Niskin bottles. Water samples were collected at three fixed depths of 50 m, 1000 m and 2000 m representing oceanic epipelagic zone biome, the oceanic mesopelagic zone biome, and the oceanic bathypelagic zone biome (deep), respectively. These CTD’s were casted in SaZr and PUZ, representing sub-Antarctic zone and polar frontal zone, respectively.

In addition to water sampling, 3 ice cores were collected at 3 stations. These ice stations were OD and IO, corresponding to open drift pancake and consolidated young sea ice, respectively. Each ice core was cut into 2 sub-sections, resulting into 2 samples per core. The sub-sectioned ice samples were then thawed at 4 °C. All melted ice core and 20 L of seawater samples were filtered sequentially onto 142 mm diameter, isopore membrane filters (Merck, USA) with nominal pore sizes of 3 µm and 0.2 µm, targeting particle-attached bacteria and large algae ( > 3 µm size fraction) and free-living bacteria and picoeukaryotes (0.2-3 µm size fraction)^83^. Subsequently, these membrane filters were flash frozen in liquid nitrogen and stored at −80ׄ°C until downstream molecular analysis.

### DMSP sample acquisition and quantification

Samples for DMSPt measurements were collected into 500 ml amber bottles from all samples described above, with each sample collected in three biological replicates. All seawater and sea ice samples were immediately treated with a proportion of 5 µl of 50 % H_2_SO_4_ per 1 ml of sample to stop biological activity and preserve DMSPt concentrations similar to^35,84^, and measurements were conducted within 30 days post expedition. Each of triplicate biological replicate samples were carefully transferred into three 20 ml GC vials (making three technical replicates), 10 ml sample was treated with 2 ml of 10 M NaOH to hydrolyze DMSP into DMS, and immediately crimped with an aluminium crimp cap, and stored in the dark until analysis^85^.

DMSPt was measured as headspace DMS, directly produced via alkaline lysis of DMSP, using a purge and trap technique of gas chromatography with flame photometric detector (GC-FPD, Agilent 7890 A GC), following the method described previously^86^. Integrated peak areas of DMS were quantified via extrapolation from an eight-point calibration curve generated from known concentrations of analytical grade DMSP (Merck) which was subjected to alkaline lysis and headspace DMS was measured, similar to^23,34,35^. DMSPt concentrations were calculated from the mean of three biological and technical replicates area peaks per sample with corresponding standard deviation. The bar plots showing DMSPt concentrations across the investigated samples were visualized using ggplot2 package^87^ implemented in R studio^88^.

### Metagenomic DNA extraction and shotgun sequencing

DNA extraction from membrane filters was performed using the Qiagen DNeasy PowerSoil Pro Kit following manufactures protocol^89^. Subsequently, the quality assurance of the extracted DNA was performed using Qubit dsDNA Assay kit in Qubit 4 Fluorometer (Thermo Fisher Scientific, USA), followed by 1 % gel electrophoresis^90^. Library preparations and metagenomics sequencing using Illumina MiSeq (2 ×150 bp) was outsourced to Admera health, USA.

### Taxonomic classification and statistics

Taxonomic classification of microbial communities from metagenomic sequences was conducted using the nr_euk database in the Kaiju classifier with default parameters^91^. The output from Kaiju was further analysed following the MicrobiotaProcess package^92,93^ to determine microbial community’s composition and relative abundances of bacteria and eukaryotes with only the top ten most abundant taxa visualized.

### Metagenomics analysis

All unprocessed metagenomes that were generated in this study are publicly available at the National Center for Biotechnology Information (NCBI). Raw reads were subjected to quality assessment using Fastqc v1.15 (https://github.com/s-andrews/FastQC) and processed using Trimmomatic v0.39 to remove adapters and low quality reads^94^. Quality filtered paired-end reads were assembled using metaSPAdes v3.15.5^95^. Assembly quality was assessed using MetaQUAST^96^. Contigs with lengths ≥ 1500 bp were used to reconstruct metagenome assembled genomes (MAGs) by mapping quality-filtered reads to assemblies using BBMap^97^ and subsequently binning using MetaBAT 2^98^. MAGs quality were assessed using CheckM v1.1.3^99^, and the genomes were classified into medium and high quality MAGs, based on previously established criteria^100^. Taxonomic classification of MAGs was inferred using GTDB-TK v2.3.0, and a maximum likelihood phylogenomic tree was constructed using the GToTree tool with default parameters^101,102^. The resultant phylogenomic tree was visualized and annotated using the iTOL v5 webserver^103^.

### Analysis of DMSP-producing and catabolizing genes

Hidden Markov Model (HMM) profiles were generated by retrieving biochemically ratified DMSP cycling protein sequences from the National Center for Biotechnology Information (NCBI)^104^. Proteins were dereplicated into non-redundant sequences using cd-hit with parameters -c0.95 -aS 80^105^. Shared sequence similarities were determined using BLASTP, followed by clustering with the Markov clustering algorithm (MCL)^106^. Clusters were aligned using MAFFT with the auto parameter implemented and trimmed using trimAl^107,108^. Trimmed alignment files were converted to HMM profiles using hmmer^109^. These profiles were used for sensitive recovery of environmental sequences retaining conserved motifs of DMSP producing and cycling enzymes using hmmscan with cutoff value -T 40% amino acid similarity and *E-value:* 1e-30 from the metagenomic dataset.

The output were normalized to the abundance of single copy housekeeping genes being *recA* for prokaryotes and 0.2 – 3 μm algae and *β-actin* for >3 μm eukaryotes^34^. Normalized counts of DMSP-producing and cycling genes were visualized for their relative abundance in tidyheatmaps v 0.1.0 implemented in R studio^110^. Taxonomic classification of DMSP producing and degrading genes were assigned using DIAMOND and implemented *E-value* 1e-50 as a cutoff parameter^111^ and TaxonKit^112^. Additionally, high quality MAGs containing selected genes encoding for DMSP synthesis (*dsyB* and *dsyGD/dsyG* and metabolism (*dddD, dddX* and *dddP*) were further investigated.

### Phylogenetic analysis of *dddP* proteins across diverse taxa

The *dddP* protein sequences were acquired from 38 medium and high-quality MAGs spanning taxonomically diverse taxa. These were aligned using MAFFT with the linsi parameter, and trimmed using trimAl with the 0.15 gap threshold^107,108^. A maximum likelihood phylogenetic tree of the resultant trimmed alignment was then reconstructed using IQ-TREE with a 1000 bootstraps^113^. Following this, phylogeny of 38 MAGs was further inferred using the GToTree tool with IQ-TREE option^102^.

### Reporting summary

Further information on research design is available in the Nature Portfolio Reporting Summary linked to this article.

## Supplementary information

Supplementary Information

Reporting Summary

## Data Availability

Unprocessed metagenomic sequence data generated in this study have been deposited in the National Center for Biotechnology Information (NCBI) under BioProject ID PRJNA1309658. Source data are provided with this paper.

## Code availability

The codes for quality control of metagenomes, genome reconstruction and HMM scan for metabolic pathways for DMSP production and degradation are available in the African-Microbiome-Project GitHub repository.
