# Supplementary material for: Dimethylsulfoniopropionate metabolism shapes microbial ecology and physiological adaptation during the austral winter in Southern Ocean sea ice and seawater
Source: Nat Commun. 2026 Jun 18;17:4864. doi: 10.1038/s41467-026-73596-x (PMC13280387; doi:10.1038/s41467-026-73596-x)
Supplement: Supplementary file 1 — Supplementary Information [file 41467_2026_73596_MOESM1_ESM.pdf]

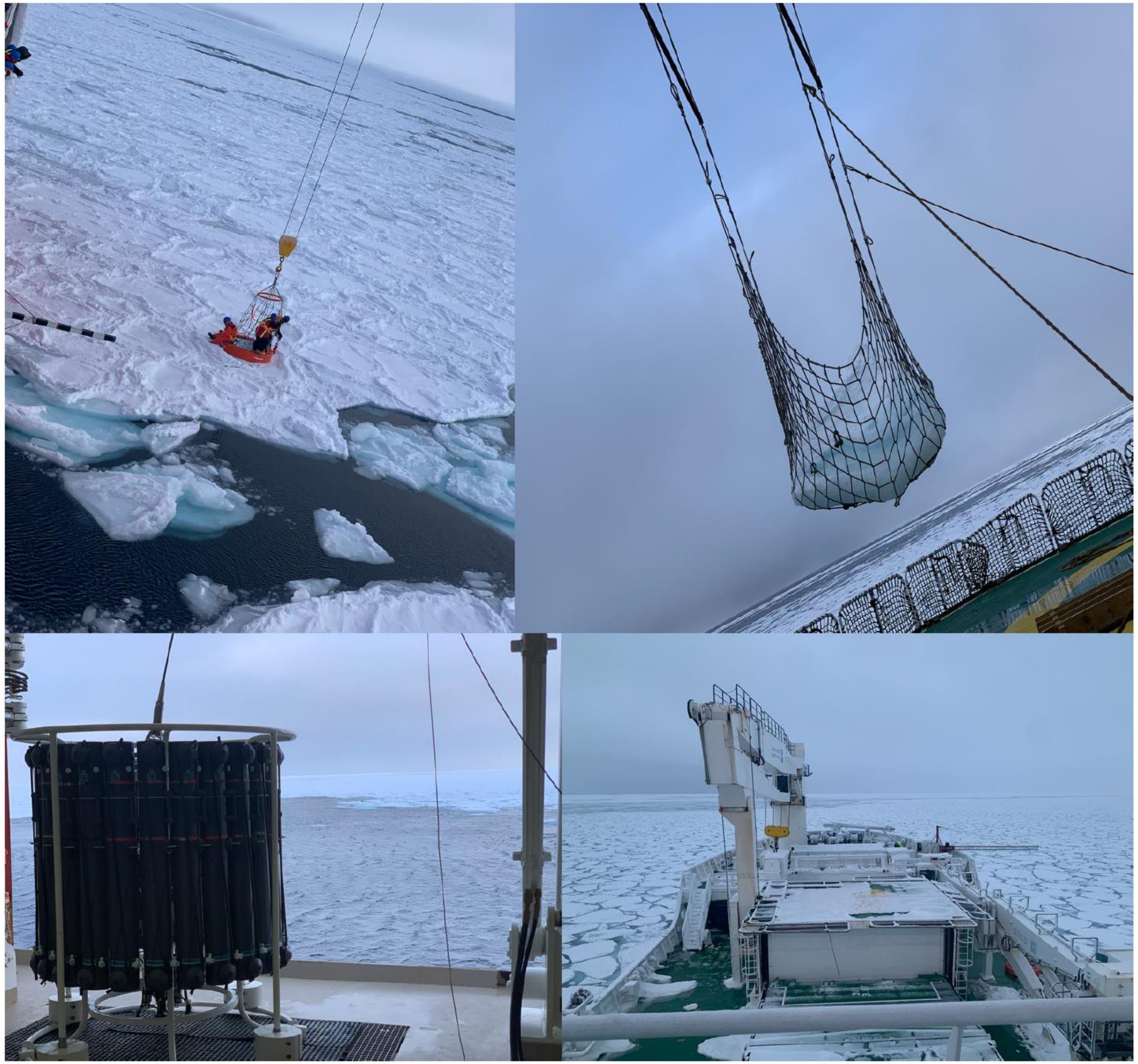

Supplementary Figure S1. Present some of the sampling stations and instruments that were used to collect seawater (Niskin bottles) and sea ice (net and insitu ice coring) in the Southern Ocean marginal ice zone.

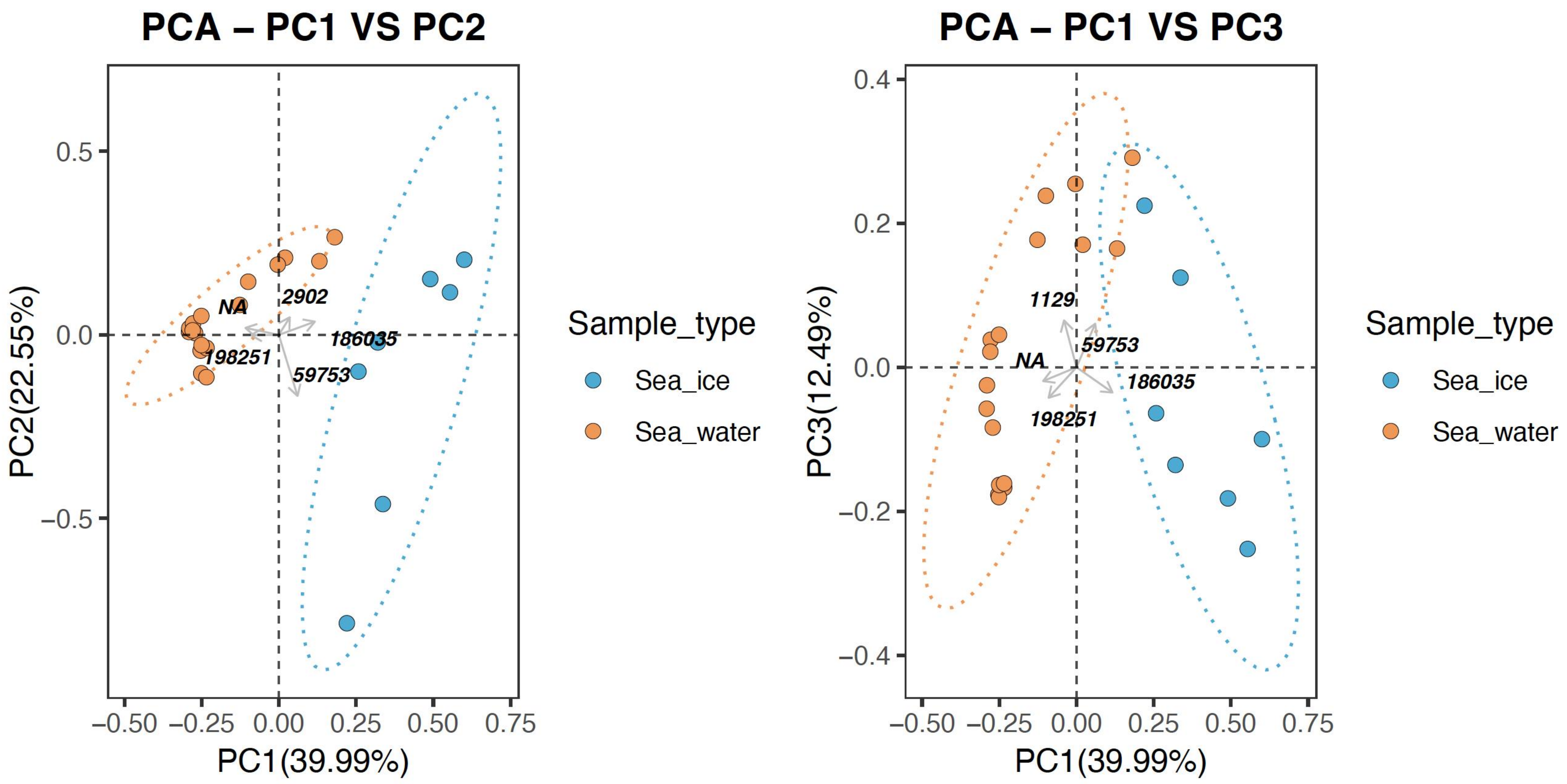

Supplementary Figure S2. Structure and diversity of microbial communities in seawater and sea ice samples based on PC1 vs PC2 and PC1 vs PC3 of the Bray–Curtis dissimilarity. Colour annotations clearly distinguish between seawater and sea ice samples.

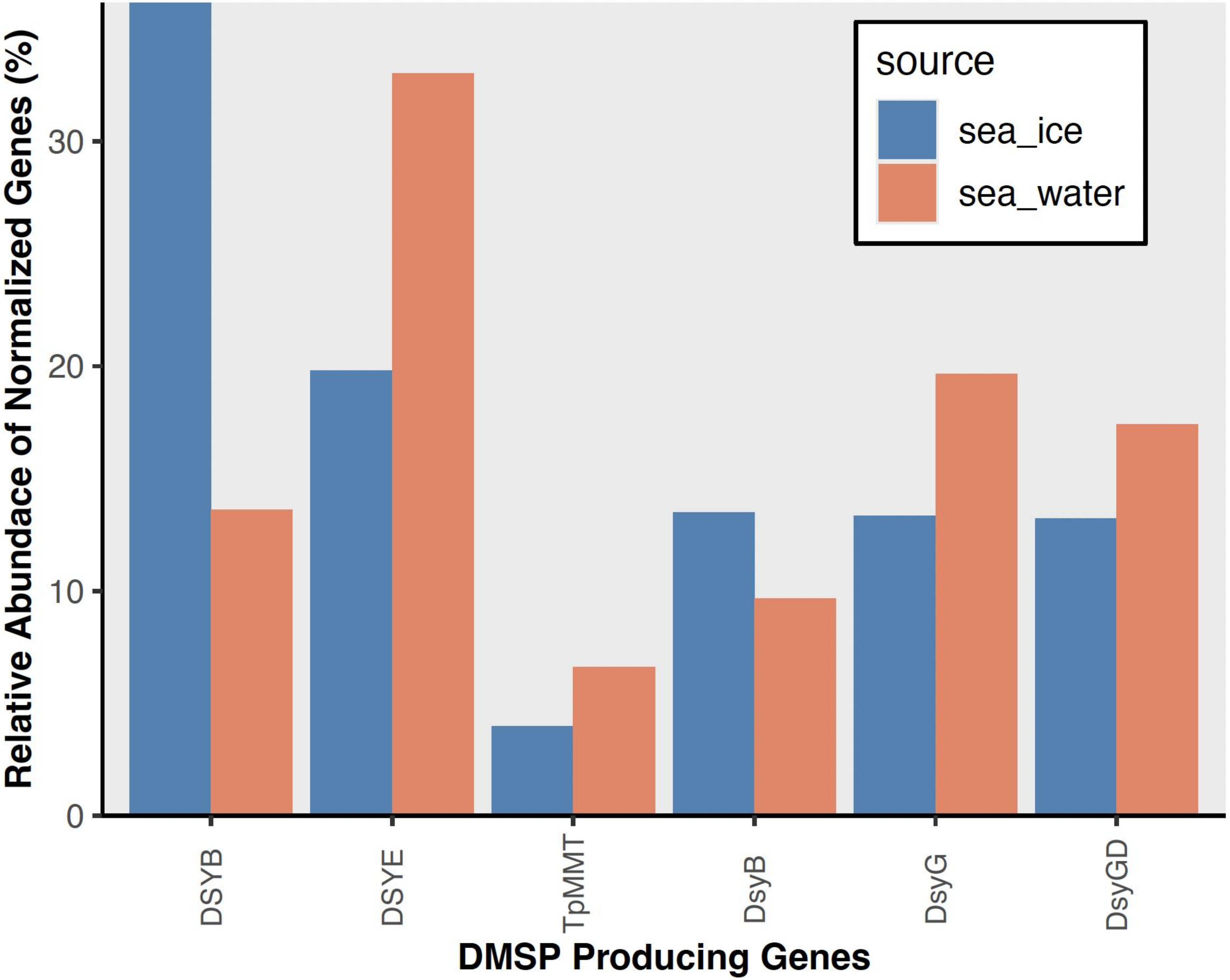

Supplementary Figure S3. Comparative relative abundance of genes encoding for microbial DMSP biosynthesis pathways between seawater and sea ice.

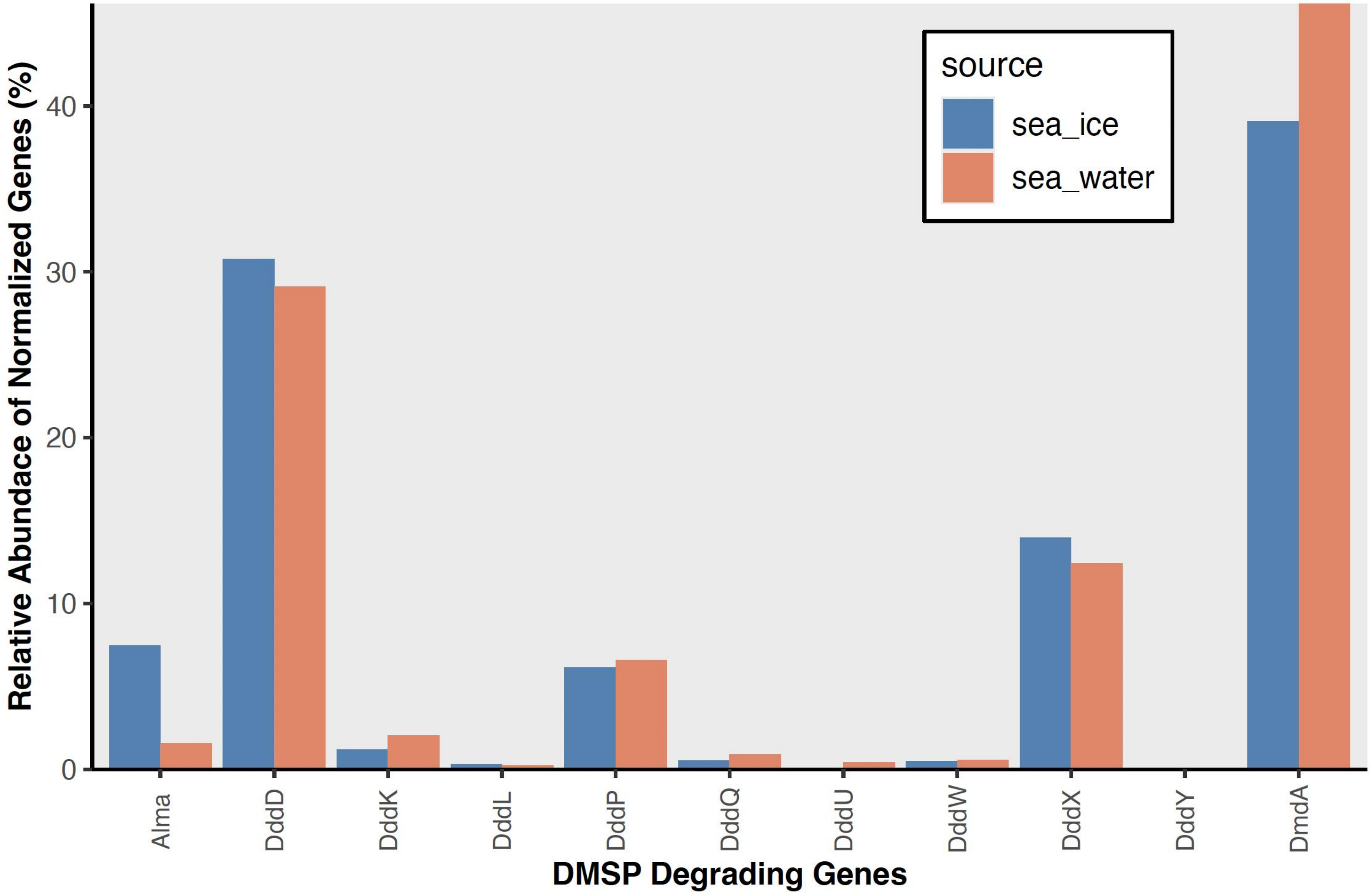

Supplementary Figure S3. Comparative relative abundance of genes encoding for microbial DMSP degradation pathways between seawater and sea ice.

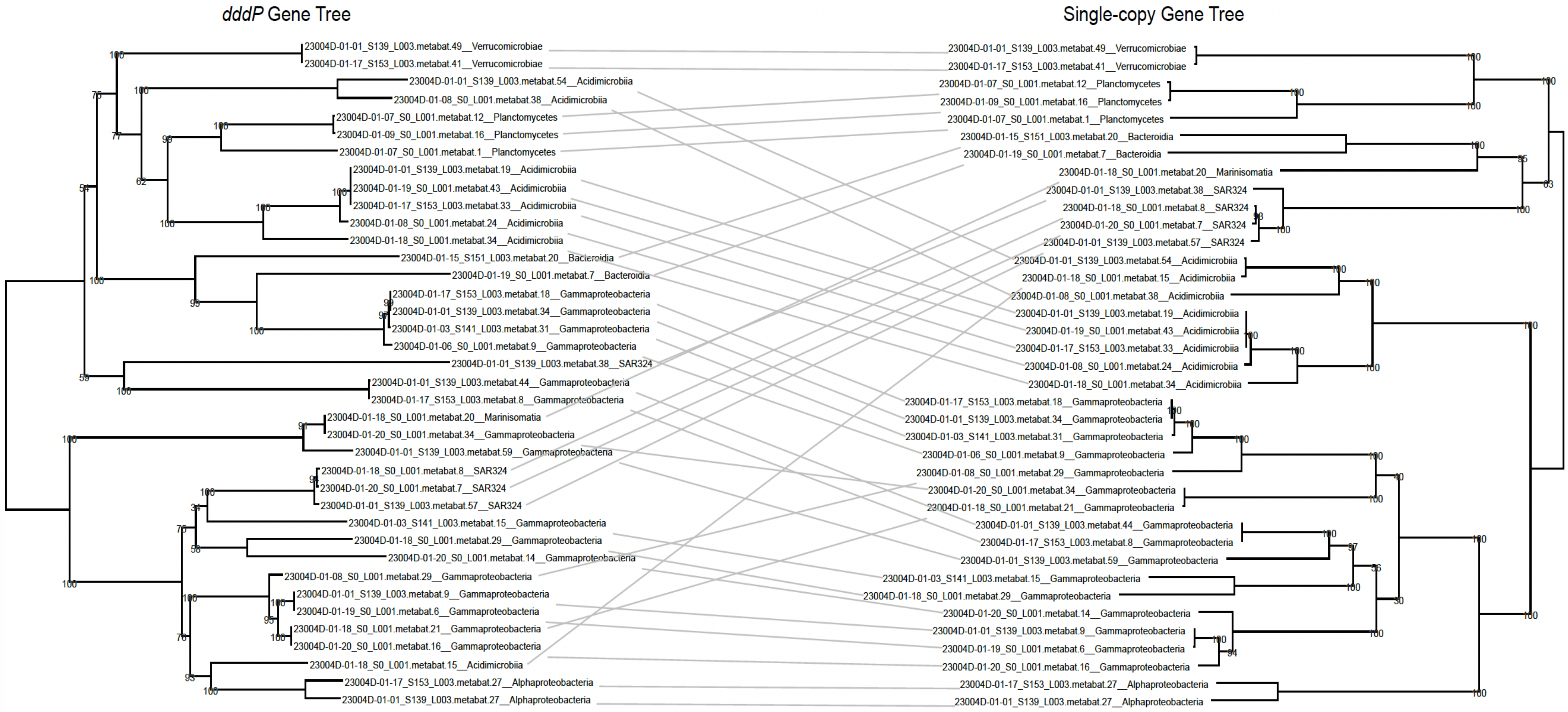

Supplementary Figure S5. Phylogenetic discordance of *dddP* proteins across diverse marine-associated taxa. The tanglegram indicates that *dddP* proteins may have undergone horizontal gene transfer. The maximum-likelihood phylogenetic tree reconstructed using *dddP* proteins is incongruent with the reference tree based on single-copy genes, underscoring the role of horizontal gene transfer in the dissemination of DMSP-cleaving genes in the ocean.
